# Enhancing the current density of a piezoelectric nanogenerator using a three-dimensional intercalation electrode

**DOI:** 10.1038/s41467-020-14846-4

**Published:** 2020-02-25

**Authors:** Long Gu, Jinmei Liu, Nuanyang Cui, Qi Xu, Tao Du, Lu Zhang, Zheng Wang, Changbai Long, Yong Qin

**Affiliations:** 1 School of Advanced Materials and Nanotechnology, Xidian University, Xi’an, 710071 China; 2 Institute of Nanoscience and Nanotechnology, Lanzhou University, Gansu, 730000 China

**Keywords:** Devices for energy harvesting, Materials for energy and catalysis, Nanowires

## Abstract

The low output current density of piezoelectric nanogenerators (PENGs) severely restricts their application for ambient mechanical energy harvest. This has been a key challenge in the development of PENG. Here, to conquer this, based on a piezoelectric material with high piezoelectric coefficient (Sm-PMN-PT), a new design of PENG with a three-dimensional intercalation electrode (IENG) is proposed. By creating many boundary interfaces inside the piezoelectric material, the total amount of surface polarization charges increased, which contributes to an increased current density. The IENG can output a maximum peak short-circuit current of 320 μA, and the corresponding current density 290 μA cm^−2^ is 1.93 and 1.61 times the record values of PENG and triboelectric nanogenerator (TENG), respectively. It can also charge a 1 μF capacitor from 0 V to 8 V in 21 cycles, and the equivalent surface charge density 1690 μC m^−2^ is 1.35 times the record value of TENG.

## Abstract (web summary)

Increasing the output current density is the key challenge for nanogenerators. Here, a new piezoelectric nanogenerator with a three-dimensional intercalation electrode is developed to reach 290 μA cm^−2^ by creating and utilizing many boundary interfaces.

## Introduction

In order to meet the urgent power supplies for multifunctional electronic devices such as portable electronic devices, implantable devices and wireless sensor networks, ambient energy harvesting technologies have developed rapidly in recent years^1^. Among them, nanogenerator including piezoelectric nanogenerator (PENG) and triboelectric nanogenerator (TENG) has attracted tremendous attentions due to their outstanding ability of converting tiny and irregular mechanical energy such as vibration, wind, walking, water wave, heartbeats and respiration movements into electricity^2,3^. Compared with TENGs, although at present, PENGs conventionally have lower output, they work more stable in extreme environment such as high moisture and dusts^4^. Hence, if the PENGs’ output can be improved to the level of TENGs, they will be one of the most powerful micro power sources.

Since the invention of PENG composed of a single ZnO nanowire (NW) deformed with an atomic force microscopy tip in 2006, great efforts have been devoted to enhancing their output performance to power the electronic devices. Up to now, the output voltage and current of PENGs have been increased from 8 mV to 250 V, and from 0.4 nA to 134 μA, respectively^5–8^. The output voltage has been rapidly increased to hundreds of volts which is high enough for most of electronic devices in our daily life, but the output current is still quite small, which has been the bottleneck of PENG’s development.

In order to improve the PENG’s output current, lots of works have been done to improve the current density of PENG by choosing materials with high piezoelectric property, such as pretreated ZnO^9^, GaN^10,11^, PVDF^12,13^, BaTiO_3_^14,15^, PbZr_0.52_Ti_0.48_O_3_^6,16^, and designing innovative device structures to realize lateral^17^, radial^18^ or vertical^9,17,19^ integrations of individual piezoelectric nanomaterials. For instance, Qin^20^ and Xu et al.^17^ grew ZnO NWs on substrates with pre-patterned seed layers by hydrothermal methods to integrate large amounts of NWs laterally and vertically, respectively, to scale up the PENGs’ current density. Through annealing, oxygen plasma and passivation pretreating process, Hu et al^9^. reduced the screening effect of free charges in ZnO NW arrays on the generated piezoelectric potential to increase the PENG’s current density. Xu et al. stacked PENGs composed of ZnO nanotip-to-nanowire arrays^21^ and PZT NW arrays^22^ in a compact way to improve the current density of PENG. Gu et al^19^. developed a method to transpose lateral aligned PZT NWs into high density vertical aligned PZT NW arrays and obtained a PENG with large current density. By these efforts, the current density was improved from 18 nA cm^−2^ in 2010^17^ to 6 μA cm^−2^ in 2011^9^, then to 23 μA cm^−2^ in 2013^19^. Later in 2014, Park et al.^6^ developed a PZT thin film-based PENG with interdigitated electrodes to connect amounts of PZT strips (width of 100 μm) parallelly, and realized a record high current density of 150 μA cm^−2^. Although big progresses have been made, the output current of PENG is still relatively small, and increasing their current density is still the key challenge.

In this work, we develop a three-dimensional intercalation electrode to enhance the current density of PENG. By creating many boundary interfaces inside the piezoelectric material, the amount of surface polarization charges is improved, and results in an increased current density. The maximum short-circuit output current reaches 320 μA, and the corresponding current density is 290 μA cm^−2^, which is 1.93 times the record value of PENG (150 μA cm^−2^)^6^ and 1.61 times the record value of TENG (180 μA cm^−2^)^23^. An IENG with effective area of only 1.2 cm^2^ can directly light up 100 red commercial LEDs. Furthermore, it can also charge a 1 μF capacitor from 0 V to 8 V in only 21 working cycles, and the equivalent surface charge density calculated from the charging process is 1690 μC m^−2^, which is 1.35 times the record value of TENG (1250 μC m^−2^)^24^.

## Results

### Characterization of Sm-PMN-PT NWs

**Fig. 1:**
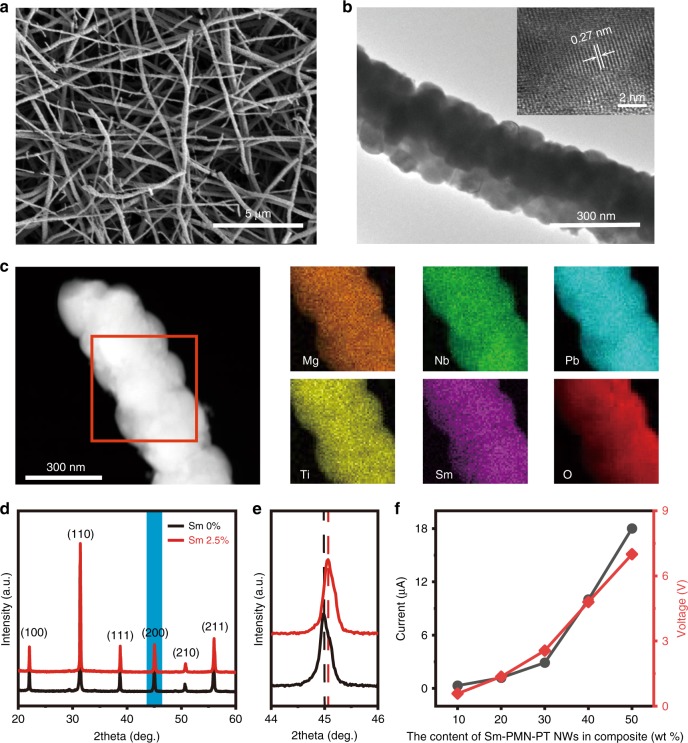
Characterization of Sm-PMN-PT NWs. **a** SEM image of annealed Sm-PMN-PT NWs. **b** Low magnification dark field image of a NW and inserted higher-magnification HRTEM image. **c** EDS mapping of a Sm-PMN-PT NW with uniform distribution of Mg, Nb, Pb, Ti, Sm and O in the NW. **d** XRD patterns of Sm doped (red) and undoped (black) PMN-PT NWs. **e** An enlarged view of the diffraction peaks around 45° marked by blue in **d**. **f** Output performance of PENGs with different content of Sm-PMN-PT NWs in the composite.

As the bulk piezoelectric coefficient of new reported Sm-PMN-PT (~1500 pC N^−1^ for polycrystalline ceramic^25^ and ~4100 pC N^−1^ for single crystal^26^) is much higher than that of traditional piezoelectric materials, such as PZT (500–600 pC N^−1^)^27^, PMN-PT (~620 pC N^−1^)^25^, so higher output current density can be expected for the Sm-PMN-PT based PENG. In this work, we first synthesize 2.5% mol Sm doped PMN-0.31PT NWs (Sm-PMN-PT NWs) by electrospinning method, and the detailed information about the preparation process can be found in the section of Methods. Fig. 1a is a representative scanning electron microscopy (SEM) image of the annealed Sm-PMN-PT NWs, from which we can find the diameter of these NWs distributes from 75 nm to 330 nm. Due to the removal of organic component and grain growth in the sintering process, the surface of these NWs is relatively rough. Transmission electron microscopy (TEM) image (Fig. 1b) shows the NW is compact and continuous. The crystal lattice spacing marked in the inset of Fig. 1b is 0.27 nm, which corresponds to the (110) crystal planes. The regular arrangement of the atoms also indicates the synthesized Sm-PMN-PT NWs have good crystallinity. A typical STEM image of Sm-PMN-PT NW and EDS qualitative element mapping are shown in Fig. 1c, it can be seen that all elements in Sm-PMN-PT are detected and distributed homogeneously in the NW. Figure 1d is the XRD patterns of the Sm doped and undoped PMN-PT NWs, which can be indexed to the polycrystalline perovskite structure. By adding 2.5 mol% Sm to replace part position of A site (Pb), the pseudocubic-tetragonal morphotropic phase boundary of PMN-*x*PT shifts from the PT content of 0.35 to 0.28^25^. Normally, the diffraction peak of Sm-PMN-PT around 45° (marked by blue area in Fig. 1d) will split into two diffraction peaks. However, the split peaks merged into one broaden peak as NWs are composed of small crystal grains, as shown in Fig. 1e. As the radius of Sm^3+^ (96 pm) is smaller than Pb^2+^ (119 pm), the diffraction pattern of Sm-PMN-PT slightly moves to the high angle compared to the undoped PMN-PT (Fig. 1e), which indicates that Sm is indeed doped into the lattice of PMN-PT. Moreover, all materials are mixed at the molecular level in the preparing process, low energy barriers of nuclei formation and diffusion lead to the synthesis of pure phase Sm-PMN-PT NWs at a low temperature of 700 °C which is much lower than the temperature in solid state sintering method^25^, as shown in Supplementary Fig. 1.

The electromechanical coupling coefficient (*d*_*33*_) of a single Sm-PMN-PT NW is measured using a piezoresponse force microscopy (PFM). A detailed description of this measurement method can be found in reference^28^. By changing the driving electric field from 0 to 10 V, a typical curve of piezoelectric displacement vs. drive voltage is recorded (Supplementary Fig. 2a). A linear fit is applied to the experimental data, and the result shows that there exists a good linearity between the piezoelectric displacement and the drive voltage. Butterfly curve and phase curve shown in Supplementary Fig. 2b demonstrate its excellent ferroelectric property. As a result, a maximum *d*_*33*_ is determined to be 142 pm V^−1^ from the slope of the linear fit. Three randomly selected NWs are tested, and total six data points are shown in Supplementary Table 1. Although the statistically averaged *d*_*33*_ value of these six data points is 120 pm V^−1^, which is about twice the reported value of PMN-PT polycrystalline NW without Sm doping (62 pm V^−1^)^28^, the value is still lower than that of their bulk form. The main reason for this phenomenon attributes to its polycrystalline structure and small grain size. Grain boundaries would significantly reduce the piezoelectric properties by obstructing domain wall movement under an external electric field and lowering the effective electric field on each grain^29^.

Benefiting from the excellent piezoelectric property of Sm-PMN-PT NW, Sm-PMN-PT NWs/PVDF composite film is suitable for fabricating PENG. Here, a Sm-PMN-PT NWs/PVDF composite film is sandwiched between the top Al film electrode and bottom Al film electrode to form a nanogenerator. During the fabrication process, a series of films with different ratios of Sm-PMN-PT NWs in Sm-PMN-PT NWs /PVDF composite (10, 20, 30, 40 and 50 wt%) are used. The corresponding average output current and voltage peak values of nanogenerators with different NWs’ content are shown in Fig. 1f. We can find that both the output current and voltage increase with the increasing of Sm-PMN-PT NWs weight percentage. The maximum output current and voltage are about 18 μA and 7 V (effective area of the device is 1.2 cm^2^). The current density of this PENG (15 μA cm^−2^) is higher than the reported maximum value of PENG made up of PVDF based composite (6.8 μA cm^−2^)^14^. A clear comparison among PVDF based PENGs is shown in Supplementary Table 2.

### Design and structure of the IENG

**Fig. 2:**
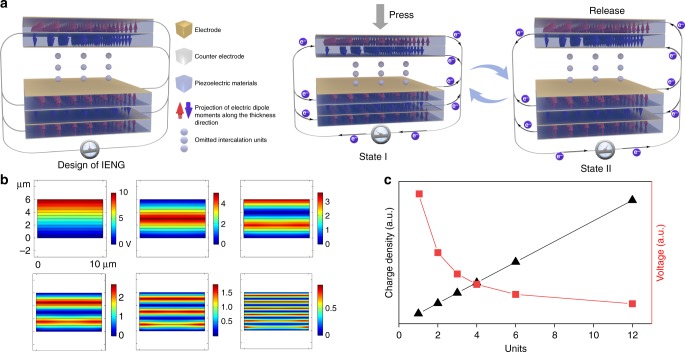
Schematics and simulation results of the IENG. **a** Basic structure of the three-dimensional intercalation electrode. Each part of piezoelectric material is sandwiched by a pair of well-matched electrodes. The adjacent parts of piezoelectric material are polarized with opposite direction, and all the parts connected in parallel forming a three-dimensional device. The red and blue arrows represent the projection of electric dipole moment along the thickness direction. When driven by an external force, each unit in the IENG simultaneously generates small current in the same direction, then adding up to be a large output current through the three-dimensional intercalation electrode. **b** Distribution of piezoelectric potential in different units when the same piezoelectric layer model is divided into 1, 2, 3, 4, 6 and 12 units by different pairs of intercalation electrode. **c** The corresponding output charge density in short-circuit condition and output voltage in open-circuit condition generated by different units.

Apart from selecting materials with high piezoelectric performance, developing the innovative structure for PENG can also contribute to the current density improvement. The latest research^30^ showed that the output current of the PENG is directly related to the Maxell’s displacement current, which can be expressed as follows:

1 $$J_Z = \frac{1}{A}\frac{{\partial (S\sigma _{P_Z})}}{{\partial t}}$$

where *J*_*z*_, *A* are the current density and cross section of the PENG, respectively. *σ*_*Pz*_, *S* are the surface polarization charge density and the corresponding boundary surface. The polarization charges only exist in the boundary surface. For the commonly used sandwich structure of PENGs, polarization charges utilized to generate output current only exist in two interfaces formed by the upper and lower electrodes with the piezoelectric material. If such interfaces are constructed substantially inside the piezoelectric material (with fixed thickness or width of *l*), the output current density may be significantly enhanced. As one of the effective ways to increase the interfaces, two-dimensional interdigital electrode has been used to improve the output current density. However, it has some shortcomings that cannot be ignored. If the electrode strips are too wide, most area of piezoelectric material are covered by electrodes, which is bad for improving the output current density. In contrast, if they are too narrow, significant nonuniformity in the resulting electric field and strains occurs, which results in internal stresses and incomplete material polarization^31^. So, the optimum width of the electrodes is a balance between the two factors, which represents the limited ability of two-dimensional interdigital electrode in constructing interfaces. To overcome the mentioned problems, a kind of PENG with a three-dimensional intercalation electrode is proposed, as shown in Fig. 2a. Compared with the case of the two-dimensional interdigital electrode, the materials are well sandwiched by pairs of electrodes, the distribution of electric field in this capacitor like structure is even, and the materials can be fully polarized even the thickness of the electrode is very thin. In addition, exerting opposite electric field directions in adjacent layers during the poling process, the adjacent layers will have opposite electric dipole moments, the projection of which along the thickness direction are indicated by red and blue arrows in Fig. 2a. Under an external force, the IENG is compressed (State I), the projection of electric dipole moments along the thickness direction becomes smaller. A small current is generated in each unit simultaneously in this process, meanwhile, all currents are added up to be a large output current through the three-dimensional intercalation electrode. When the force disappears, the pressure releases and all electric dipole moments return to their original state (State II), resulting in a reversed output current. In this case, the induced surface polarization charges in the boundaries add up without cancelation to increase the IENG’s current density. To quantitatively investigate the influence of three-dimensional intercalation electrode on PENG’s output performance, a theoretical model is constructed and calculated using COMSOL Multiphysics software, as shown in Fig. 2b. The piezoelectric block with size of 10 μm × 6 μm is treated as the cross section of a three-dimensional device. In the simulation, the piezoelectric layer is divided into 1, 2, 3, 4, 6 and 12 same units by different pairs of intercalation electrode, and adjacent parts obtain opposite polarization direction. Under the same compressive force, the potential distribution in open-circuit condition and the total charge density in short-circuit condition are calculated. The statistical results in Fig. 2c show that more units for a fixed thickness of piezoelectric layer will generate higher charge density (corresponding to high current density) and lower voltage.

**Fig. 3:**
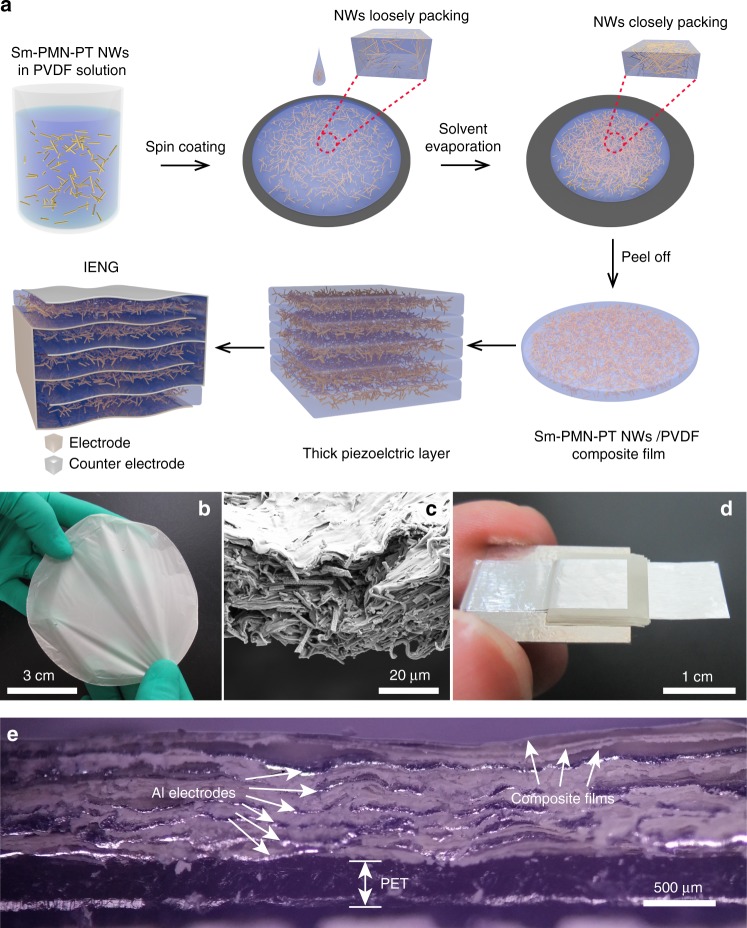
Fabrication and characterization of the IENG. **a** Schematic diagram of the fabrication process for IENG. **b** Optical image of a round Sm-PMN-PT/PVDF composite film with diameter of 9 cm. **c** Cross-section image of the composite film in **b**. **d** Optical image of a fabricated IENG with 7 units. Al electrodes on each side is connected as one electrode forming a three-dimensional intercalation electrode. **e** The corresponding cross-section image of IENG. Al electrodes, composite films and PET are marked by white arrows.

As shown in Fig. 3a, based on the above design, the IENG with a three-dimensional intercalation electrode is developed. As dividing a whole piezoelectric layer into many parts with same thickness is a very difficult process, so we choose an opposite way in which the piezoelectric layers are constructed by fixed number of fabricated piezoelectric thin films. Firstly, the solution of PVDF and Sm-PMN-PT NWs is prepared by mixing them uniformly in N, N-Dimethylformamide (DMF) and acetone. Then the solution is spin-coated on a Si substrate and cured on a hot plate at 120 °C for 10 min. After the evaporation of solvents (acetone and DMF), the state of Sm-PMN-PT NWs in PVDF shifts from loosely packing to closely packing due to shrinking of the composite film. Secondly, the composite thin film peels off from the Si substrate. The optical photograph of a round composite film with 9 cm diameter is given in Fig. 3b. The fabrication process is simple, cost effective and suitable for large-scale production. The thickness of the composite film is about 30 μm, as shown in Fig. 3c. Thirdly, the top and bottom sides of the composite thin film are encapsulated with the precured PDMS-coated Al (Al/PDMS) electrodes. After these steps, one PENG unit composed of one piece of piezoelectric thin film is fabricated. Lastly, the IENG with multiple units is realized by stacking more single units. Fig. 3d shows an optical image of IENG with 7 units. All the Al/PDMS electrodes on the right side and left side are connected together, respectively, to form the three-dimensional intercalation electrode. Multi-unit structure is attached on a PET substrate (thickness of 300 μm), and the Al/PDMS electrodes (thickness of 15 μm) are marked by white arrows as shown in Fig. 3e. For this device, there are three composite films between two adjacent Al/PDMS electrodes, and the average thickness of each unit is about 110 μm.

### Output performance of IENG

**Fig. 4:**
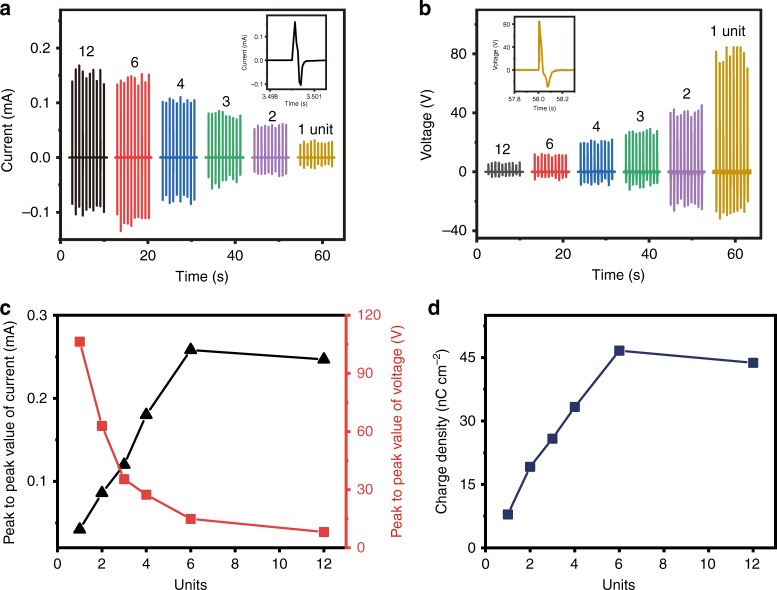
Output performance of IENGs with different units. Piezoelectric layer is constructed by 12 composite thin films, and then divided into 1, 2, 3, 4, 6 and 12 units by different pairs of intercalation electrode. Adjacent units obtain opposite polarization direction. **a**, **b** Output current and output voltage of each case. Insets are the enlarged views of output current generated by 12 units and output voltage by 1 unit, respectively. **c** The trend of peak to peak values of output current and voltage in **a** and **b** with the increase of units. **d** The trend of charge densities calculated from current signals with the increase of units.

Using the Sm-PMN-PT NWs/PVDF composite thin film as the basic piezoelectric material (50 wt% Sm-PMN-PT NWs), six IENGs with same thickness of piezoelectric layer (all composed by 12 piezoelectric thin films), but with different number of units (1, 2, 3, 4, 6 and 12) are fabricated and characterized. Due to pairs of electrodes are different in each device, the thicknesses of six IENGs are about 390, 405, 420, 435, 465 and 555 μm, respectively. The output current and voltage of these devices are shown in Fig. 4a, b. With the units decreasing from 12 to 6, there is little difference between the output currents, both are about 150 μA. And then the current decreases from 150 μA to 25 μA with the units decreasing from 6 to 1. Differently, the voltage increases from 6 V to 84 V with the units decreasing from 12 to 1. As the output current and voltage above are all AC signals, their average peak to peak values could reflect the trends more obviously. Furthermore, for a NG with AC output signals, the output charge density that can be defined as the ratio of output charges generated by a positive (or negative) peak to active area, is also an important parameter to reflect its power output capability. From the results as shown in Fig. 4c, d, we can find that the voltage decreases nonlinearly with the increase of the number of units, which is consistent with the simulation result (Fig. 2c). The main reason for this trend is that the output voltage of PENG is closely related to the thickness of piezoelectric layer. For the current and charge density, they present an approximate linear relationship with the increasing of units from 1 to 6, which is consistent with simulation result (Fig. 2c). But both of the average peak to peak value of current and charge density decrease slightly and deviate from the simulation result when the units increased from 6 to 12. In the previous simulation, the Al/PDMS electrode is not considered. Instead, zero electric field displacement, zero potential boundary condition for interface of adjacent units were used to simulate the open-circuit and short-circuit behavior of the IENGs, respectively. So, the influence of Al/PDMS electrodes on the output performance of IENGs is not taken into account. Moreover, more units mean more pairs of Al/PDMS electrodes in the device. The ratio of electrodes to piezoelectric thin films in 6 and 12 units in this part are 7/12 and 13/12, respectively. High ratio of soft Al/PDMS electrodes in the device may generate stronger buffering effect, resulting in smaller strain in the piezoelectric layer. As a consequence, the current and charge density begin to decrease.

### Performance enhancement and application of the IENG

**Fig. 5:**
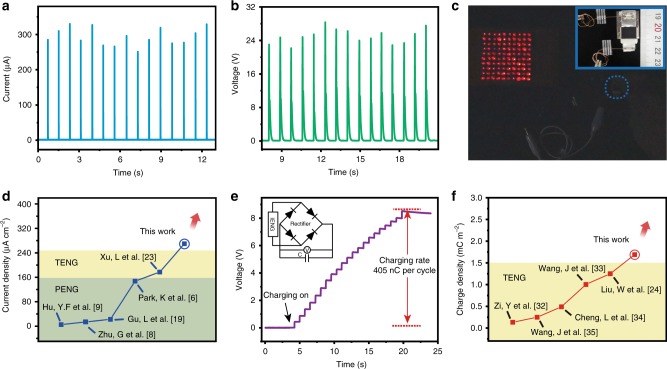
Output performance of the IENG and its position in reported works. **a**, **b** The rectified current and voltage signals. **c** 100 red LEDs are instantaneously lighted up by the IENG without storage process. Inserted image shows optical image of the IENG, and the black rubber on it is used to uniformly disperse the stress. **d** The maximum current density of our IENG calculated from rectified current signals in **a** and some representative PENGs and TENG. **e** Charging curve for a 1 μF capacitor, and the corresponding charging rate is 405 nC per cycle. **f** Charge density of our IENG calculated from the charging curve in **e** and selected representative TENGs.

Based on the simulation and experiment results above, an IENG with ultrahigh current density and proper output voltage are fabricated. In the device, each unit is composed of three composite thin films due to its output voltage (~20 V) is enough for most applications. Then a series of IENGs with different units are fabricated (1, 3, 6, 12 and 24), and the thicknesses of these devices are about 110, 330, 660, 1320 and 2640 μm, respectively. With the units increasing from 1 to 24, the current output increases from 18 μA to about 270 μA (Supplementary Fig. 3a). Supplementary Figure 3b shows the statistical average peak values and the corresponding theoretical charge densities calculated from Supplementary Fig. 3a. With an active area of 1.2 cm^2^, the maximum output charges in single peak (each cycle has two peaks) can reach 120 nC under stress of 0.06 MPa. To demonstrate a practical high-power application of IENG, we connect it to a commercial light-emitting diode (LED) arrays consisting of 100 diodes through a bridge rectifier. The rectified output voltage and current are displayed in Fig. 5a, b, respectively. Under stress of 0.1 MPa, the maximum output current and voltage can reach 329 μA and 28 V. 100 commercial red LEDs (10 LEDs are connected in series to form a LED strip, and then 10 such LED strips are further connected in parallel forming the LED arrays) with operation voltage of 1.8 V can be directly and simultaneously lighted up without storage process as shown in Fig. 5c and Supplementary Movie 1. Image inserted in Fig. 5c shows the real IENG device, and the black rubber is used to uniformly disperse the stress. In this case, the maximum current density is 290 μA cm^−2^, which is 1.93 times the record of PENG^6^ and 1.61 times the record of TENG^23^. A clear comparison among PENGs is shown in Supplementary Table 3. Figure 5d shows the current densities of representative PENGs^6,8,9,19^ and TENG^23^, we can find that the current density of our work is a new record. Up to now, as the electricity generated by PENG or TENG needs to be stored in a capacitor or battery for most application cases, the output charges will be more appropriate than current density to characterize the NG’s output performance. Hence, a charging curve for a 1 μF capacitor is measured to obtain the actual charging rate of our IENG (Fig. 5e). We can see that the capacitor can be charged to about 8 V in 21 working cycles of IENG, the corresponding average charging rate is 405 nC per cycle. As most work about PENG do not report the output charge density, so we make a comparison of the output charge density between our IENG and reported TENGs. An ultrahigh output charge density of 1690 μC m^−2^ is calculated from the charging curve of our IENG in Fig. 5e, which is larger than the reported values of TENGs^24,32–35^ and 1.35 times the record one^24^ (Fig. 5f).

**Fig. 6:**
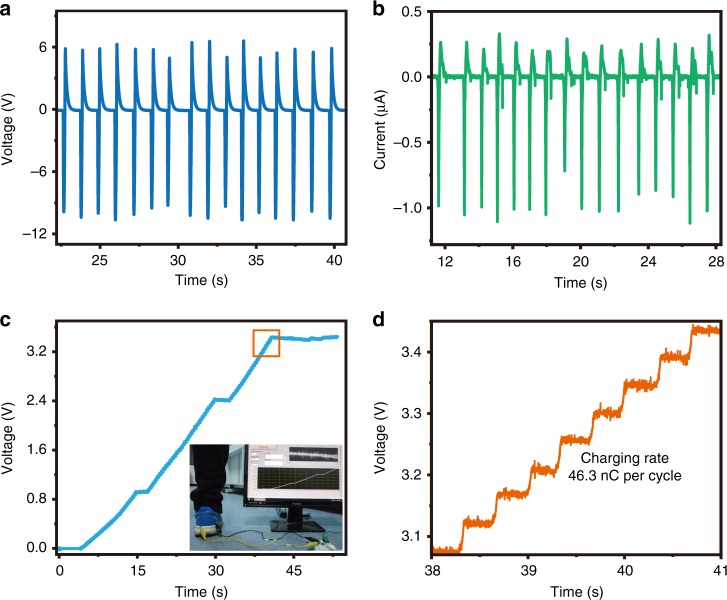
Application of the IENG on harvesting biomechanical energy. **a**, **b** Output voltage and output current signals of IENG driven by human walking. **c** The corresponding charging curve for a 1 μF capacitor after rectified. Inserted image shows real scene of charging process. **d** An enlarged view of the area marked by orange square in **c**. The corresponding charging rate is 46.3 nC per cycle.

As human-motion-charged power source has wide applications in realizing self-powered portable electronic systems, the IENG is well designed to efficiently harvest the biomechanical energy from human walking. In the experiment, the IENG is directly placed under the insole cushion. Foot stepping (~60 N) is used to drive our IENG (the area of heel is about 50 cm^2^, the corresponding stress applied on the IENG is about 0.012 MPa), the obtained voltage and current signals are about 10 V (Fig. 6a) and 1 μA (Fig. 6b). After rectifying, a storage curve for a 1 μF capacitor is shown in Fig. 6c, and the inset is an optical image of the charging process. The whole charging process can be seen in the Supplementary Movie 2. Fig. 6d is an enlarged view of the region marked by orange square in Fig. 6c, from which an average charging rate of 46.3 nC per cycle can be calculated, and the corresponding charge density is 193 μC m^−2^. The output performance here is lower than that in Figs. 4 and 5, which is mainly due to the smaller stress and lower stress rate caused by the cushioning effect of insole cushion.

## Discussion

In summary, based on Sm-PMN-PT polycrystalline NW with a high piezoelectric coefficient of 120 pm V^−1^, a new design of PENG with a three-dimensional intercalation electrode is proposed to increase the PENG’s output current density. By constructing many boundary interfaces inside the piezoelectric materials, large amounts of surface polarization charges are induced, and the output current of IENG is increased. Its maximum output current reaches 329 μA, and the corresponding current density 290 μA cm^−2^ is 1.93 times the record value of PENG and 1.61 times the record value of TENG. For an IENG with effective area of 1.2 cm^2^, its output could directly light up 100 red commercial LEDs, and an ultrahigh surface charge density (1690 μC m^−2^) that is 1.35 times the biggest one is realized. In addition, the IENG is successfully used to efficiently harvest biomechanical energy from human walking, and the output charge density is 193 μC m^−2^. This work will push forward the development of nanogenerator and has great potential in realizing self-powered electronic systems.

## Methods

### Synthesis of Sm-PMN-PT NWs by electrospinning method

By adding samarium nitrate hexahydrate (Sm(NO_3_)_3_·6H_2_O) to the PMN-PT precursor recipe reported previously^28^, Sm-PMN-PT nanofibers are fabricated. Lead acetate trihydrate (Pb(C_2_H_3_O_2_)_2_·3H_2_O), samarium nitrate hexahydrate (Sm(NO_3_)_3_·6H_2_O), magnesium ethoxide (Mg(OC_2_H_5_)_2_), niobium ethoxide (Nb(OC_2_H_5_)_5_) and titanium isopropoxide (Ti(OCH(CH_3_)_2_)_4_) are used as source materials. Acetylacetonate (AcAc) is used as complexing agent. Firstly, stable Sm-PMN-PT complex precursor is synthesized through distillation and refluxing of the starting materials in 2-methoxyethanol under dry nitrogen-gas atmosphere. To compensate for lead loss during rapid thermal annealing, 5 mol % excess lead is added to the precursor solution. Secondly, PVP with molecular weight of 1300,000 is dissolved into 2-methoxyethanol, and then added into the precursor under vigorously stirring to form a homogenous precursor solution. Thirdly, the well-mixed solution is loaded into the plastic syringe and pumped through the needle at a speed of 0.1 mL min^−1^. The needle is connected with the positive electrode of the power source, and the collecting plate is connected with ground. The distance between the needle and the collecting plate is 15 cm. A high voltage of 18 kV is applied to achieve a stable Tylor cone. Al foil is used to collect NWs. Finally, the as-spun NWs are annealed in a furnace at 850 °C to achieve perovskite structure.

### Fabrication of Sm-PMN-PT samples for PFM measurement

Firstly, a silicon substrate (0.5 cm^2^) is coated with a layer of Pt by magnetron sputtering. Secondly, Sm-PMN-PT NWs are deposited on the Pt coated substrate, then annealed at 700 °C to achieve the perovskite structure and closely contacted with the substrate. Thirdly, the Pt layer is connected with a metal sample mount using silver paste and then grounded.

### Fabrication of IENG

Considering that dividing a whole piezoelectric material into many parts with same thickness is very difficult, here we choose an opposite way in which the piezoelectric material is constructed by fixed number of easy-fabricated piezoelectric thin films. Firstly, Sm-PMN-PT NWs/PVDF composite thin films are prepared by spin-coating method (2000 r min^−1^), and then cured on a hot plate at 120 °C for 10 min. Secondly, the composite thin film peels off from the Si substrate and cuts into required size. Thirdly, Al foil as an electrode is fixed on a PET substrate (~300 μm). Fourthly, a layer of PDMS (~2 μm) is spin-coated on Al electrode and cured on a hot plate at 100 °C for 15 min forming the Al/PDMS electrode. Fifthly, the composite thin film is attached to Al/PDMS electrode tightly through the strong adhesion of PDMS. Sixthly, another layer of PDMS is spin-coated on the composite thin film. Seventhly, Al foil as the counter electrode is attached to the composite thin film. By now, a device with one unit is fabricated. IENG with different units can be obtained by repeating the fourth, fifth and sixth steps.

### Measurement

All IENGs in this work are polarized under a 5 kV mm^−1^ electric field at 110 °C for 30 min before the electric measurement. A round plastic stick with diameter of 1.5 cm connected with the linear motor (LinMot E1100) is used to periodically drive the IENGs, meanwhile, a pressure sensor below IENG is used to detect the pressure. Low noise preamplifiers (SR570, SR560) are used to measure the output voltage and current, and PCI-6259 (National Instruments) is used for data collection. A software platform based on LabVIEW is used to realize real-time data acquisition and analysis.

## Supplementary information

Supplementary Information

Description of Additional Supplementary Files

Supplementary Movie 1

Supplementary Movie 2

## Data Availability

The data that support the findings of this study are available from the corresponding author upon reasonable request.
