# Supplementary material for: Enhancing the current density of a piezoelectric nanogenerator using a three-dimensional intercalation electrode
Source: Nat Commun. 2020 Feb 25;11:1030. doi: 10.1038/s41467-020-14846-4 (PMC7042353; doi:10.1038/s41467-020-14846-4)
Supplement: Supplementary file 1 — Supplementary Information [file 41467_2020_14846_MOESM1_ESM.pdf]

Enhancing the current density of a piezoelectric nanogenerator using a three-dimensional intercalation electrode

Long Gu[1,†], Jinmei Liu[1,†], Nuanyang Cui[1], Qi Xu[1], Tao Du[1], Lu Zhang[1], Zheng Wang[1], Changbai Long[1] and Yong Qin[,2]*

[1] School of Advanced Materials and Nanotechnology, Xidian University, Xi'an, 710071, China;

[2] Institute of Nanoscience and Nanotechnology, Lanzhou University, Gansu, 730000, China.

[†] Authors with equal contribution.

* Corresponding author E-mail: qinyong@lzu.edu.cn.

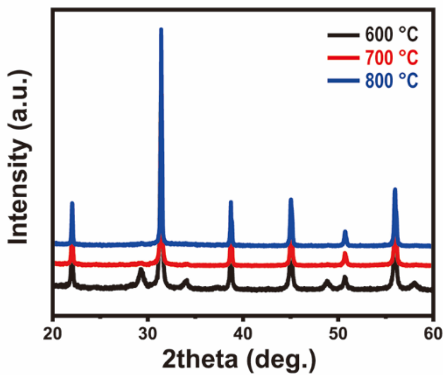

Supplementary Figure 1 | XRD patterns of Sm-PMN-PT NWs sintered under different temperatures.

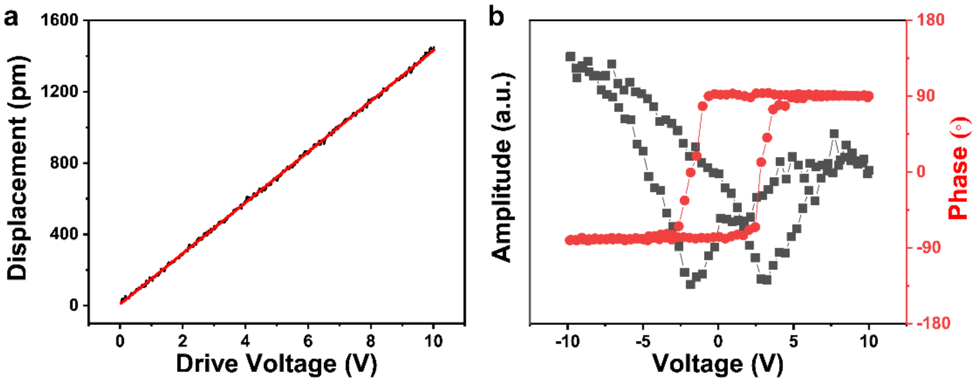

Supplementary Figure 2 | **a**, Piezoelectric displacement vs voltage curve of a Sm-PMN-PT nanowire. The black line is experiment data, and the red one is PFM data. The slope of fitted curve shows the piezoelectric coefficient d_{33} is about 142 pm V^{-1}. **b**, Butterfly curve and phase curve of Sm-PMN-PT nanowire.

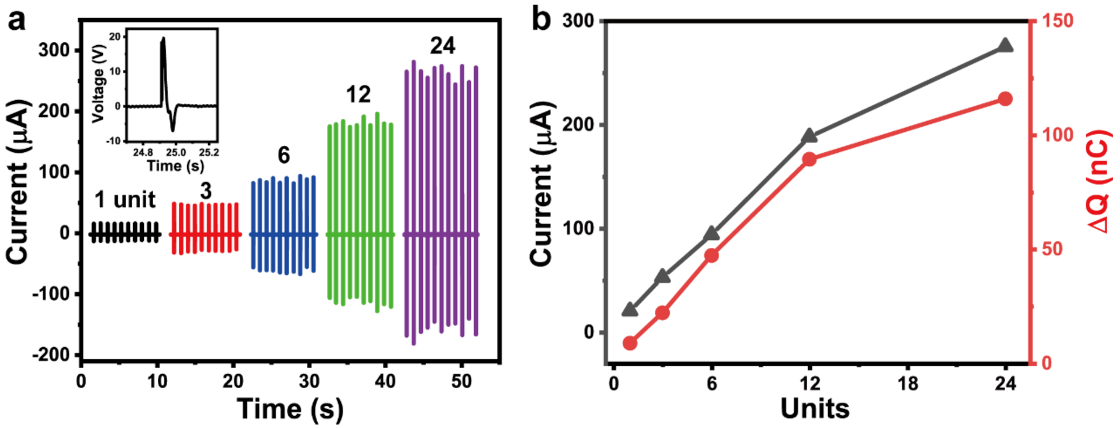

Supplementary Figure 3 | Output performance of IENGs with different units. Each unit is composed by three composite films. **a**, Output currents of IENGs with 1, 3, 6, 12 and 24 units. Inset is an enlarged view of output voltage generated by IENG with 24 units. **b**, The trend of average current peak values and the corresponding charge densities calculated from output current in **a** with different units.

Supplementary Table 1 | *d_{33}* values from different Sm-PMN-PT nanowires and locations. The statistical average value from these data is 120.23 pm V^{-1}.

Data point	Slope	d_{33}（pm V^{-1}）
NW1, point 1	0.115725	115.73
NW1, point 2	0.119134	119.00
NW2, point 1	0.116673	116.67
NW2, point 2	0.103807	103.80
NW3, point 1	0.123786	123.79
NW3, point 2	0.142382	142.38

Supplementary Table 2 | The output performance of PVDF based PENGs.

Materials	Current	Current density	Voltage	Reference
PVDF	70 nA	145 nA cm^{-2}	3 V	1
	300 nA	/	1.5 V	2
PVDF nanofibers	3 nA	/	10 mV	3
	60 nA	/	2 V	4
	250 nA	40 nA cm^{-2}	2.5 V	5
	/	/	1.5 V	6
PVDF-TrFE	58 nA	0.56 μA cm^{-2}	7 V	7
	/	0.8 μA cm^{-2}	4 V	8
	2.6 μA	/	4 V	9
PVDF/rGO	0.92 μA	/	90 V	10
$FAPbBr_3$/PVDF	/	6.2 μA cm^{-2}	30 V	11
PVDF/$BaTiO_3$	200 nA	/	0.7 V	12
	2.4 μA	/	6.7 V	13
	1.5 μA	/	150 V	14
	15 μA	6.8 μA cm^{-2}	75 V	15
PVDF/BCZT	/	0.17 μA cm^{-2}	42 V	16
PVDF/$NaNbO_3$	4.4 μA	/	3.4 V	17
PVDF/PMN-PT	14.5 μA	12.1 μA cm^{-2}	5.2 V	this work
PVDF/Sm-PMN-PT	18 μA	15 μA cm^{-2}	7 V	this work
PVDF/Sm-PMN-PT with a 3D intercalation electrode	320 μA	290 μA cm^{-2}	30 V	this work

Supplementary Table 3 | The output performance of PENGs based on different materials.

Materials	Current	Current density	Voltage	Power density	Reference
GaN	40 nA	0.16 μA cm^{-2}	1.2 V	/	18
GaN/V_2O_5	850 nA	/	21 V	/	19
ZnO	107 nA	/	2.03 V	11 mW cm^{-3}	20
	6 μA	6 μA cm^{-2}	20 V	0.2 W cm^{-3}	21
	134 μA	15 μA cm^{-2}	58 V	0.78 W cm^{-3}	22
ZnO/P3HT	/	8.13μA cm^{-2}	2.2 V	0.88 W cm^{-3}	23
ZnO/Cu_xO	/	11.4μA cm^{-2}	26 V	/	24
MoS_2	20 pA	/	15 mV	2 mW m^{-2}	25
BN	200 nA	22.2 nA cm^{-2}	9 V	0.03 μW cm^{-2}	26
PVDF	70 nA	145 nA cm^{-2}	3 V	/	1
PVDF-TrFE	2.6 μA	/	4 V	5.0 μW cm^{-2}	9
$BaTiO_3$	26 nA	0.19 μA cm^{-2}	1.0 V	7 mW cm^{-3}	27
	350 nA	350 nA cm^{-2}	5.5 V	/	28
PZT	0.55μA	/	20 V	/	29
	53 μA	23.5 μA cm^{-2}	209 V	/	30
	180 μA	150 μA cm^{-2}	200 V	/	31
$ZnSnO_3$	0.13 μA	/	5.3 V	11 μW cm^{-3}	32
$NaNbO_3$	72 nA	16 nA cm^{-2}	3.2 V	0.6 mW cm^{-3}	33
$KNbO_3$	67.5 nA	9.3 nA cm^{-2}	3.2 V	/	34
PVDF/rGO	0.92 μA	/	90 V	0.34 mW cm^{-3}	10
BCZT	/	0.17 μA cm^{-2}	42 V	161.7 mW m^{-2}	16
BSFTO	2.8 μA	0.62 μA cm^{-2}	16 V	3.11 μW cm^{-2}	35
PMN-PT	2.29μA	4.58 μA cm^{-2}	7.8 V	/	36
	14.5 μA	12.1 μA cm^{-2}	5.2 V	/	this work
Sm-PMN-PT	18 μA	15 μA cm^{-2}	7 V	/	this work
Sm-PMN-PT with a 3D intercalation electrode	320 μA	290 μA cm^{-2}	30 V	/	this work

Supplementary References

1. Jin, L. et al. Polarization-free high-crystallization β-PVDF piezoelectric nanogenerator toward self-powered 3D acceleration sensor. *Nano Energy* **50**, 632-638 (2018).
2. Cha, S. et al. Porous PVDF as effective sonic wave driven nanogenerators. *Nano Lett.* **11**, 5142-5147 (2011).
3. Chang, C., Tran, V. H., Wang, J., Fuh, Y. K. & Lin, L. Direct-write piezoelectric polymeric nanogenerator with high energy conversion efficiency. *Nano Lett.* **10**, 726-731 (2010).
4. Fuh, Y. K., Chen, P. C., Huang, Z. M. & Ho, H. C. Self-powered sensing elements based on direct-write, highly flexible piezoelectric polymeric nano/microfibers. *Nano Energy* **11**, 671-677 (2015).
5. Fuh, Y. K., Kuo, C. C., Huang, Z. M., Li, S. C. & Liu, E. R. A transparent and flexible graphene-piezoelectric fiber generator. *Small* **12**, 1875-1881 (2016).
6. Wang, X. et al. Bionic single-electrode electronic skin unit based on piezoelectric nanogenerator. *ACS Nano* **12**, 8588-8596 (2018).
7. Pi, Z. Y., Zhang, J. W., Wen, C. Y., Zhang, Z. B. & Wu, D. P. Flexible piezoelectric nanogenerator made of poly(vinylidenefluoride-co-trifluoroethylene) (PVDF-TrFE) thin film. *Nano Energy* **7**, 33-41 (2014).
8. Lee, J. H. et al. Highly sensitive stretchable transparent piezoelectric nanogenerators. *Energy Environ. Sci.* **6**, 169-175 (2013).
9. Chen, X. et al. A high performance P(VDF-TrFE) nanogenerator with self-connected and vertically integrated fibers by patterned EHD pulling. *Nanoscale* **7**, 11536-11544 (2015).
10. Bhunia, R. et al. Milli-watt power harvesting from dual triboelectric and piezoelectric effects of multifunctional green and robust reduced graphene oxide/P(VDF-TrFE) composite flexible films. *ACS Appl. Mater. Interfaces* **11**, 38177-38189 (2019).
11. Ding, R. et al. High-performance piezoelectric nanogenerators composed of formamidinium lead halide perovskite nanoparticles and poly(vinylidene fluoride). *Nano Energy* **37**, 126-135 (2017).
12. Guo, W. et al. Wireless piezoelectric devices based on electrospun PVDF/$BaTiO_3$ NW nanocomposite fibers for human motion monitoring. *Nanoscale* **10**, 17751-17760 (2018).
13. Hu, P. H., Yan, L. L., Zhao, C. X., Zhang, Y. Y. & Niu, J. Double-layer structured PVDF nanocomposite film designed for flexible nanogenerator exhibiting enhanced piezoelectric output and mechanical property. *Compos. Sci. Technol.* **168**, 327-335 (2018).
14. Zhao, Y. L. et al. High output piezoelectric nanocomposite generators composed of oriented $BaTiO_3$ NPs@PVDF. *Nano Energy* **11**, 719-727 (2015).
15. Shin, S. H., Kim, Y. H., Lee, M. H., Jung, J. Y. & Nah, J. Hemispherically aggregated $BaTiO_3$ nanoparticle composite thin film for high-performance flexible piezoelectric nanogenerator. *ACS Nano* **8**, 2766-2773 (2014).
16. Wu, Y., Qu, J., Daoud, W. A., Wang, L. & Qi, T. Flexible composite-nanofiber based piezo-triboelectric nanogenerators for wearable electronics. *J. Mater. Chem. A* **7**, 13347-13355 (2019).
17. Zeng, W. et al. Highly durable all-fiber nanogenerator for mechanical energy harvesting. *Energy Environ. Sci.* **6**, 2631-2638 (2013).
18. Lin, L. et al. High output nanogenerator based on assembly of GaN nanowires.

Nanotechnology **22**, 475401 (2011).

19. Waseem, A. et al. Effect of crystal orientation of GaN/V_2O_5 core-shell nanowires on piezoelectric nanogenerators. *Nano Energy* **60**, 413-423 (2019).
20. Zhu, G., Yang, R., Wang, S. & Wang, Z. L. Flexible high-output nanogenerator based on lateral ZnO nanowire array. *Nano Lett.* **10**, 3151-3155 (2010).
21. Hu, Y., Lin, L., Zhang, Y. & Wang, Z. L. Replacing a battery by a nanogenerator with 20 V output. *Adv. Mater.* **24**, 110 (2012).
22. Zhu, G., Wang, A. C., Liu, Y., Zhou, Y. & Wang, Z. L. Functional electrical stimulation by nanogenerator with 58 V output voltage. *Nano Lett.* **12**, 3086-3090 (2012).
23. Lee, K. Y. et al. P-type polymer-hybridized high-performance piezoelectric nanogenerators. *Nano Lett.* **12**, 1959-1964 (2012).
24. Johar, M. A., Kang, J. H., Ha, J. S., Lee, J. K. & Ryu, S. W. Controlled conductivity of p-type Cu_xO/GaN piezoelectric generator to harvest very high piezoelectric potential. *J. Alloys Compd.* **726**, 765-771 (2017).
25. Wu, W. et al. Piezoelectricity of single-atomic-layer MoS_2 for energy conversion and piezotronics. *Nature* **514**, 470-474 (2014).
26. Lee, G. J. et al. Piezoelectric energy harvesting from two-dimensional boron nitride nanoflakes. *ACS Appl. Mater. Interfaces* **11**, 37920-37926 (2019).
27. Park, K. I. et al. Piezoelectric $BaTiO_3$ thin film nanogenerator on plastic substrates. *Nano Lett.* **10**, 4939-4943 (2010).
28. Lin, Z. H. et al. $BaTiO_3$ nanotubes-based flexible and transparent nanogenerators. *J. Phys. Chem. Lett.* **3**, 3599-3604 (2012).
29. Chou, X. et al. All-in-one filler-elastomer-based high-performance stretchable piezoelectric nanogenerator for kinetic energy harvesting and self-powered motion monitoring. *Nano Energy* **53**, 550-558 (2018).
30. Gu, L. et al. Flexible fiber nanogenerator with 209 V output voltage directly powers a light-emitting diode. *Nano Lett.* **13**, 91-94 (2013).
31. Park, K. I. et al. Highly-efficient, flexible piezoelectric PZT thin film nanogenerator on plastic substrates. *Adv. Mater.* **26**, 2514-2520 (2014).
32. Wu, J. M. et al. Flexible and transparent nanogenerators based on a composite of lead-free $ZnSnO_3$ triangular-belts. *Adv. Mater.* **24**, 6094-6099 (2012).
33. Jung, J. H. et al. Lead-free $NaNbO_3$ nanowires for a high output piezoelectric nanogenerator. *ACS Nano* **5**, 10041-10046 (2011).
34. Jung, J. H. et al. Lead-free $KNbO_3$ ferroelectric nanorod based flexible nanogenerators and capacitors. *Nanotechnology* **23**, 375401 (2012).
35. Zhang, Y. et al. Performance enhancement of flexible piezoelectric nanogenerator via doping and rational 3D structure design for self-powered mechanosensational system. *Adv. Funct. Mater.* **29** (2019).
36. Xu, S. et al. Flexible piezoelectric PMN-PT nanowire-based nanocomposite and device. *Nano Lett.* **13**, 2393-2398 (2013).
