# Supplementary material for: Enhancing the current density of a piezoelectric nanogenerator using a three-dimensional intercalation electrode
Source: Nat Commun. 2020 Feb 25;11:1030. doi: 10.1038/s41467-020-14846-4 (PMC7042353; doi:10.1038/s41467-020-14846-4)
Supplement: Supplementary file 2 — Description of Additional Supplementary Files [file 41467_2020_14846_MOESM2_ESM.pdf]

Description of Additional Supplementary Files

File Name: Supplementary Movie 1
Description: 100 red LEDs instantaneously lighted up by IENG without storage process.

File Name: Supplementary Movie 2
Description: Harvest the energy of human walking using IENG.
